# Complete coding sequence of two feline panleukopenia virus strains isolated from domestic cats (*Felis catus*) in Tennessee, USA

**DOI:** 10.1128/MRA.00431-23

**Published:** 2023-09-28

**Authors:** Ashkan Roozitalab, Ola K. Elsakhawy, Mohamed A. Abouelkhair

**Affiliations:** 1 Department of Biomedical and Diagnostic Sciences, University of Tennessee, Knoxville, Tennessee, USA; Katholieke Universiteit Leuven, Leuven, Belgium

**Keywords:** feline panleukopenia virus, parvoviridae, carnivore protoparvovirus 1, feline parvovirus, feline parvoviral enteritis, virology, veterinary microbiology

## Abstract

Here, we announce the complete coding sequence of two strains of feline panleukopenia virus (FPLV) that were obtained from deceased domestic cats in animal shelters in Tennessee. The provided sequence data will contribute to a deeper comprehension of the genetic characteristics and evolutionary patterns of FPLV in the USA.

## ANNOUNCEMENT

Feline panleukopenia virus (FPLV), belonging to the *Parvovirus* genus, is a non-enveloped, single-stranded DNA virus that infects a wide range of animals beyond domestic cats, including raccoons, tigers, lions, and minks, making it a significant concern for both domestic and wild feline populations (1–5). It causes acute gastroenteritis and leukopenia, particularly in cats that have not been vaccinated (6, 7). FPLV is highly contagious and spreads easily between cats, especially in shelters and catteries (7, 8).

Two loops of small intestinal tissues were collected from deceased cats in 2022. Subsequently, one gram of the collected tissue was homogenized in 200 µL of phosphate-buffered saline using sterile glass beads for 15 minutes and subsequently filtered using a sterile 0.22-µM syringe filter. The resulting filtrate was then used to inoculate a monolayer of Crandell-Rees feline kidney (CRFK) cells (ATCC, CCL-94) (9). The total DNA was then extracted from infected CRFK cells using MagMAX Viral/Pathogen Nucleic Acid Isolation Kit (Thermo Fisher Scientific, USA). The NanoDrop 2000 spectrophotometer and Qubit fluorometer were used to assess the quality and quantity of extracted DNA. Using the complete genome of feline parvovirus (FPV) (MT614366) as the reference, the forward primer (5′-GAATGATAGGCGGTTTGTGTGT-3′) and reverse primer (5′-CCTACGCGGTCTGGTTGATT-3′) were designed to amplify the coding sequence of FPVL using Geneious Prime (10).

The coding sequence of FPV was amplified using the LongAmp Taq 2× Master Mix (New England Biolabs, USA), following the manufacturer’s instructions. The amplicons were purified using GenCleanTurbo kit (MP Biomedicals, USA)

PCR amplicon libraries were prepared using the Nextera DNA Flex kit (Illumina, Inc., USA) following the manufacturer’s instructions. Two FPLV strains were sequenced using Illumina MiniSeq (150 bp × 2) (Illumina, Inc.) resulting in 621,221 and 604,347 high-quality reads for each VI2882 and VI2534 strains, respectively. The raw reads for each sequence were trimmed using BBDuk followed by mapping to reference by Geneious Prime version 2023.0.1. Default parameters were used for all software unless otherwise specified (10). The National Center for Biotechnology Information open reading frame (ORF) finder (https://www.ncbi.nlm.nih.gov/orffinder/) was used to predict each ORF using default parameters.

**Fig 1:**
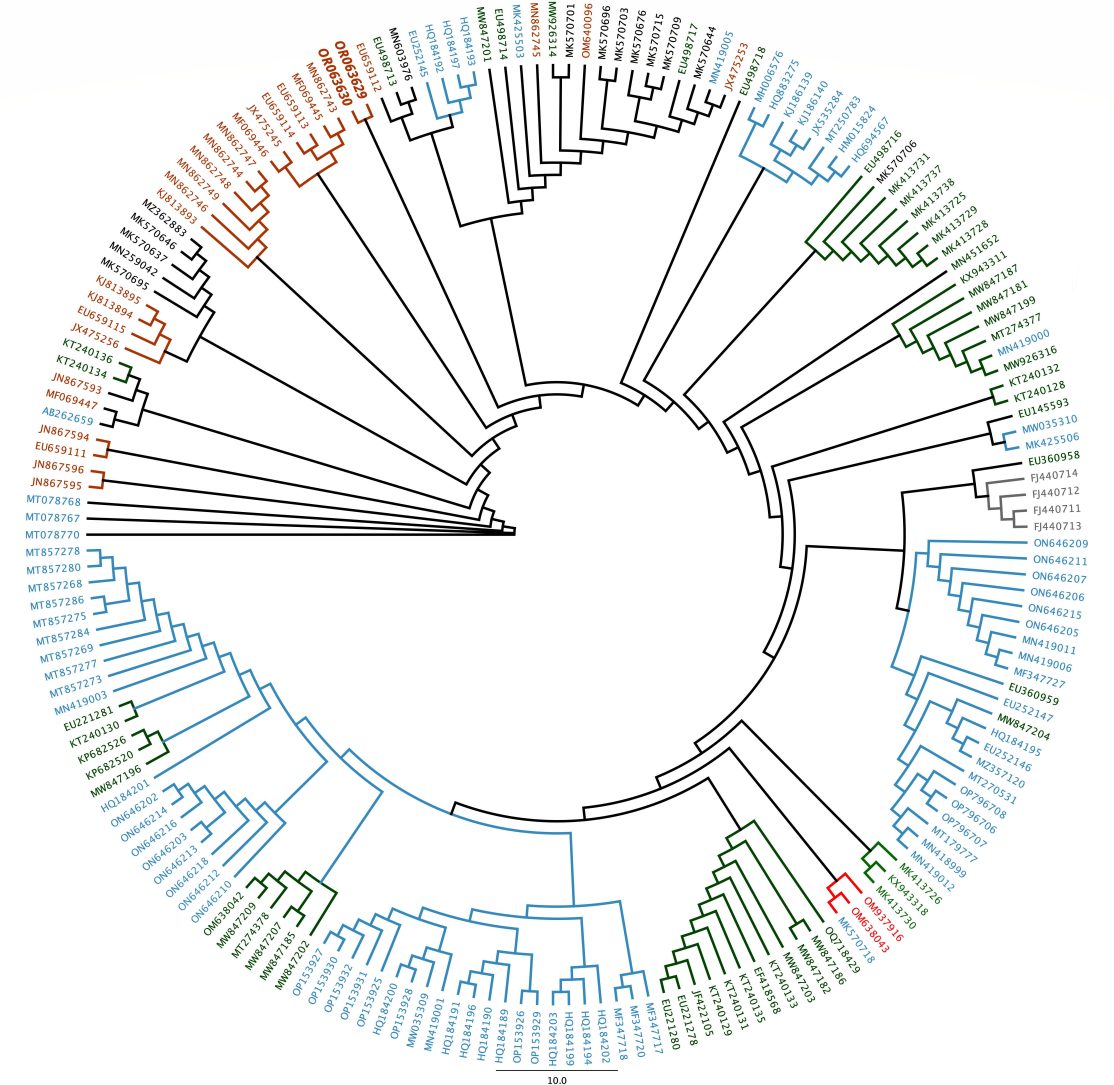
Maximum-likelihood tree showing the genetic relationship of the full-length *VP2* gene of feline panleukopenia viruses. The strains that were sequenced in this study, which are denoted in bold and italic, formed a cluster within the FPVL strains isolated from domestic cats in North America. The sequences shown in orange correspond to strains isolated from North America, while blue koi indicates strains from Asia; dark green represents strains from Europe; black designates strains from Oceania; red from Africa; and gray from South America.

The complete coding sequence of strains VI2882 and VI2534, spanning nearly the entire genome, consists of 4,269 nucleotides. Moreover, the mean read coverage for VI2882 and VI2534 isolates was 34,117 and 28,327, respectively. The guanine-cytosine (GC) contents for VI2882 and VI2534 were 36.2% and 35.9%, respectively. The full-length *VP2* gene of VI 2882 and VI 2534 FPVL strains, along with feline parvovirus strains from various continents, was retrieved from the National Center for Biotechnology Information virus (11) (Fig. 1). The sequences were aligned using Clustal Omega version 1.2.3 (12, 13). Phylogenetic relationships among FPVL strains based on *VP2* sequences were analyzed using maximum-likelihood method through PHYML version 3.2.20180621 (14). The strains subjected to sequencing in this study clustered together with FPVL strains isolated from domestic cats in North America.

## Data Availability

The complete coding sequence of the strains has been deposited in GenBank under accession numbers OR063629 and OR063630. The National Center for Biotechnology Information's (NCBI's) Sequence Read Archive (SRA) accession numbers are available under SRS17591485 and SRS17591484.
